# Stretchable electronic strips for electronic textiles enabled by 3D helical structure

**DOI:** 10.1038/s41598-024-61406-7

**Published:** 2024-05-14

**Authors:** Jessica Stanley, Phil Kunovski, John A. Hunt, Yang Wei

**Affiliations:** 1 Smart Wearable Research Group, Department of Engineering, Nottingham Trent University, Nottingham, UK; 2 Medical Technologies Innovation Facility, Nottingham Trent University, Nottingham, UK; 3 Kymira Ltd, Reading, UK; 4 College of Biomedical Engineering, China Medical University, Taichung, 40402 Taiwan

**Keywords:** Electrical and electronic engineering, Electronic devices

## Abstract

The development of stretchable electronic devices is a critical area of research for wearable electronics, particularly electronic textiles (e-textiles), where electronic devices embedded in clothing need to stretch and bend with the body. While stretchable electronics technologies exist, none have been widely adopted. This work presents a novel and potentially transformative approach to stretchable electronics using a ubiquitous structure: the helix. A strip of flexible circuitry (‘e-strip’) is twisted to form a helical ribbon, transforming it from flexible to stretchable. A stretchable core—in this case rubber cord—supports the structure, preventing damage from buckling. Existing helical electronics have only extended to stretchable interconnects between circuit modules, and individual components such as printed helical transistors. Fully stretchable circuits have, until now, only been produced in planar form: flat circuits, either using curved geometry to enable them to stretch, or using inherently stretchable elastomer substrates. Helical e-strips can bend along multiple axes, and repeatedly stretch between 30 and 50%, depending on core material and diameter. LED and temperature sensing helical e-strips are demonstrated, along with design rules for helical e-strip fabrication. Widely available materials and standard fabrication processes were prioritized to maximize scalability and accessibility.

## Introduction

### Stretchable electronics for electronic textiles

Stretchable electronics technology is a key component in the development of electronic textiles (e-textiles), where electronic devices are embedded in clothing and other textiles^1,2^. This is because many textiles are stretchable, including those used in sportswear, where clothing must not restrict the wearer’s movement. Or medical compression garments, where tight-fitting stretch fabric is used to apply pressure^3^. As healthcare and sports are key markets for e-textiles^4,5^, there is a need for reliable, stretchable e-textiles. And while there are existing methods to fabricate stretchable electronics^6^, none has yet emerged as standard. Sewing techniques used to make seams in stretch garments use zigzag or other looping stitches that extend as the garment stretches, and these have been used to stitch stretchable conductive thread tracks on fabric^7^. However, while it is possible to attach components directly to conductive threads, this is challenging^8^, and conductive threads are primarily used as interconnects between rigid circuit modules^9–11^ and flexible modules^12,13^ or to construct textile sensors^14–16^. Conductive threads are breathable and conform well to fabric, but have limited washability^17^.

Similarly, flexible electronic circuits (normally meaning metallic tracks printed or etched on flexible polymer substrates, optionally containing rigid components) may be formed in serpentine/horseshoe shapes. For example, cutting a polyimide (PI) substrate into a serpentine geometry, allowing the whole assembly to stretch^18^. Or forming serpentine metal tracks on a stretchable substrate such as thermoplastic polyurethane (TPU), polydimethylsiloxane (PDMS), or other elastomers^19,20^. However, these materials have limited compatibility with many standard electronics manufacturing processes, for example deforming when exposed to the high (> 200 °C) temperatures normally required for soldering, the most common and durable method to attach components to conductive tracks. The main disadvantage of serpentine tracks is that stresses concentrate in certain fixed areas^21,22^, which ultimately leads to failure caused by cracking in metal tracks.

Another way to achieve stretchable electronics is to use controlled buckling, where conductive fibers or ribbons are embedded in a pre-strained elastomer substrate. Releasing the substrate, and allowing it to contract, causes the embedded conductor to bend or fold into a stretchable geometry. Existing work includes out-of-plane buckling to make stretchable ribbons^23^, and in-plane buckling to realize stretchable interconnects and LED circuits, using a fiber drawing approach^24^.

Another, less common, approach to stretchable e-textiles borrows from the Japanese art of kirigami, creating stretchable conductive textiles by strategically placing cuts in fabric ^25^, or creating kirigami-inspired nanomaterials^26^. Another example combines a folded origami structure with kirigami elements^27^. Stretchable printed electronics can also be fabricated using stretchable conductive inks^28^ or liquid metal^29^, but these are less common and typically exhibit high variation in resistance when stretched, so they are mostly used for strain sensing.

In this work, we employed a helical or spring geometry, ubiquitous in both natural and engineered structures. Existing work using helical geometry in electronics has focused on helical interconnects joining planar circuit modules, rather than entire circuits. This includes helical polyurethane (PU) and copper fibers^30^, helices of nanoparticle coated yarn embedded in PDMS^31^, helical conductive yarn^32^, helical copper interconnects embedded in silicone^33^, and helical interconnects for epidermal electronics^34^. Controlled buckling has also been used to create form microwires into helical electrodes embedded in elastomers^24^. Individual helical components have also been created, including knitted inductors^35^, helical ‘fiber pumps’^36^ and printed helical transistors^37^. Helical electrodes formed from copper wire wrapped around a nylon core have also been developed for use in robotic skin^38^, as well as helical energy harvesting devices^39^, and flexible batteries using helical electrodes^40^. Braiding has also been used to create a helical structure from optic fibers and conductive yarns for touch sensing, though this is not a stretchable structure^41^.

These demonstrated the potential of helical geometry for stretchable electronics, but fully helical circuits using components have not yet been explored. This is perhaps surprising, as when a helix extends it distributes stress evenly along its structure, which could be highly beneficial for preventing failures in stretchable electronics. This work proposes a fabrication process for fully helical circuits (“helical e-strips”). A key part of this is the use of a long strip of flexible circuitry, rather than a conductive fiber, wire or yarn, to allow established PCB manufacturing processes (including pick and place machines to populate components on circuits, and reflow soldering) to be used, instead of processes that require manual soldering of components. This can facilitate circuits with more than one or two traces to be realized in helical form, without increasing the complexity of the circuit assembly process, and enable scalability.

**Figure 1:**
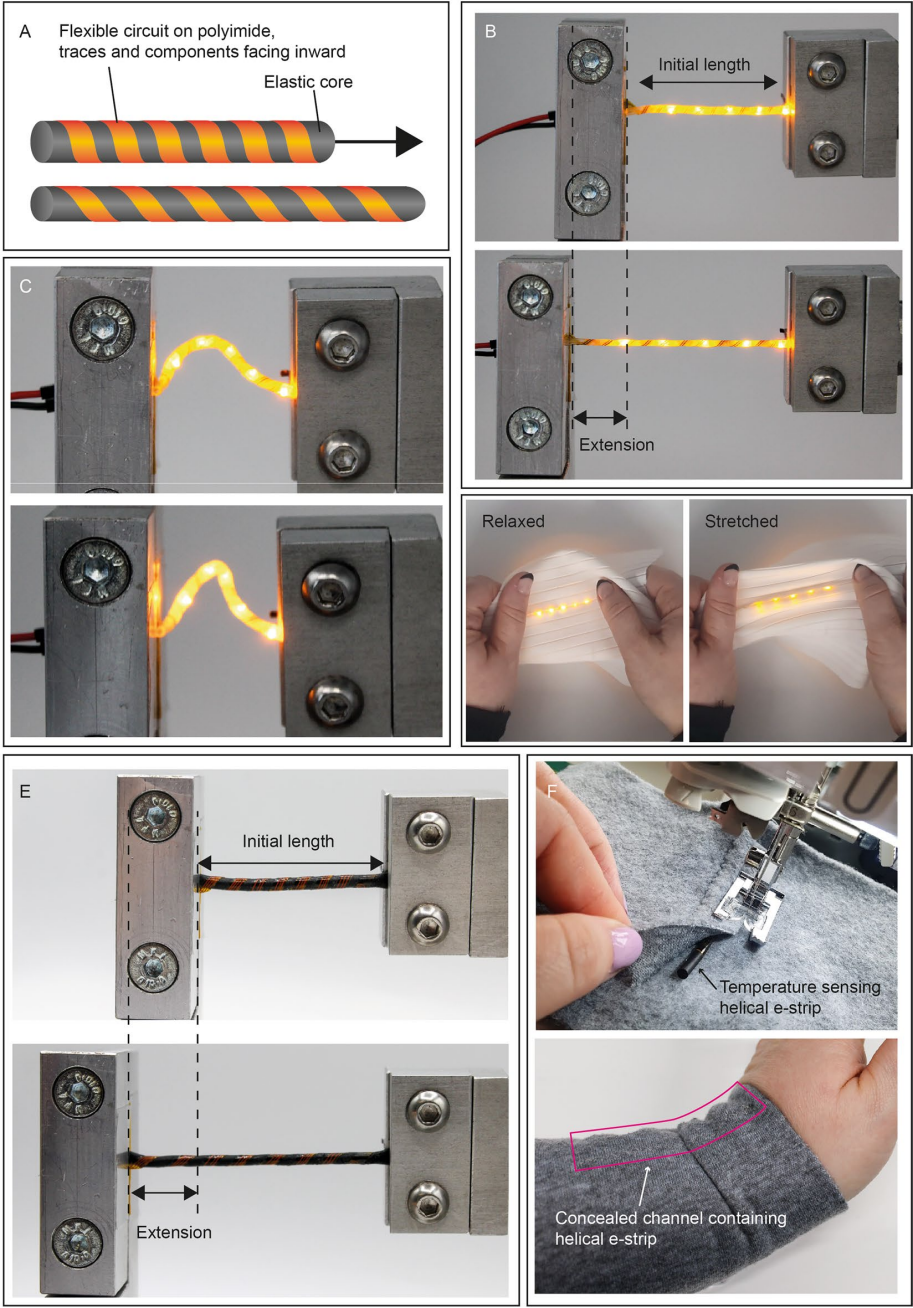
Helical e-strip concept: (**A**) illustration of fundamental concept; (**B**) Demonstration of stretchability of a helical LED e-strip; (**C**) Demonstration of flexibility of helical LED e-strip; (**D**) Example helical e-strip application: stretchable illuminated fabric; (**E**) Demonstration of helical sensing e-strip stretchability; (**F**) Example application: stitching a helical temperature sensing e-strip into fabric to measure body temperature, barely visible in the finished sleeve. Additional images in Supplementary Information Fig. 1.

This method was first tested on blank helical e-strips with no components or conductive tracks, to determine design rules for fabrication. And to determine optimal geometry for maximizing stretch, while minimizing overall diameter. Next, conductive tracks were added to form helical interconnect e-strips and subjected to tensile tests. Then, helical LED e-strips were demonstrated and evaluated through tensile and wash-cycle testing. Finally, helical temperature sensing e-strips were fabricated, and their performance compared to a planar, flexible temperature sensor. Most fabrication steps used standard processes used in flexible electronics manufacturing, minimizing the need for custom equipment and materials, enabling an easy transition from prototyping to manufacturing. The concept and demonstrations of helical e-strip stretchability are presented in Fig. 1.

For all electronic parts intended for use in e-textiles, it is important to consider integration into textiles. Helical e-strips are designed for insertion into concealed channels in garments, rather than knitting or weaving into the textile structure (Fig. 1D,F). A similar method has been used to house flexible electronic modules in e-textiles, e.g., Wicaksono et al.^18^ demonstrated a custom knit garment with built-in channels designed to house stretchable electronic strips. This method has also been used with woven fabrics^42^, or fabric channels stitched onto an existing garment, into which electronic parts were inserted^7^. This keeps electronic and textile components of the e-textile garment separate until final assembly, and allows electronics to be removable for repair or recycling, in line with sustainability recommendations^43,44^. Helical e-strips could also be knit or woven into the fabric, as has been demonstrated with planar, flexible electronic strips^45^. Other options include attaching e-strips to garments using tailored fiber placement^46^, or lamination^47^, but the specific question of textile integration was not the focus of this work.

## Results

### Helical e-strip composition

**Figure 2:**
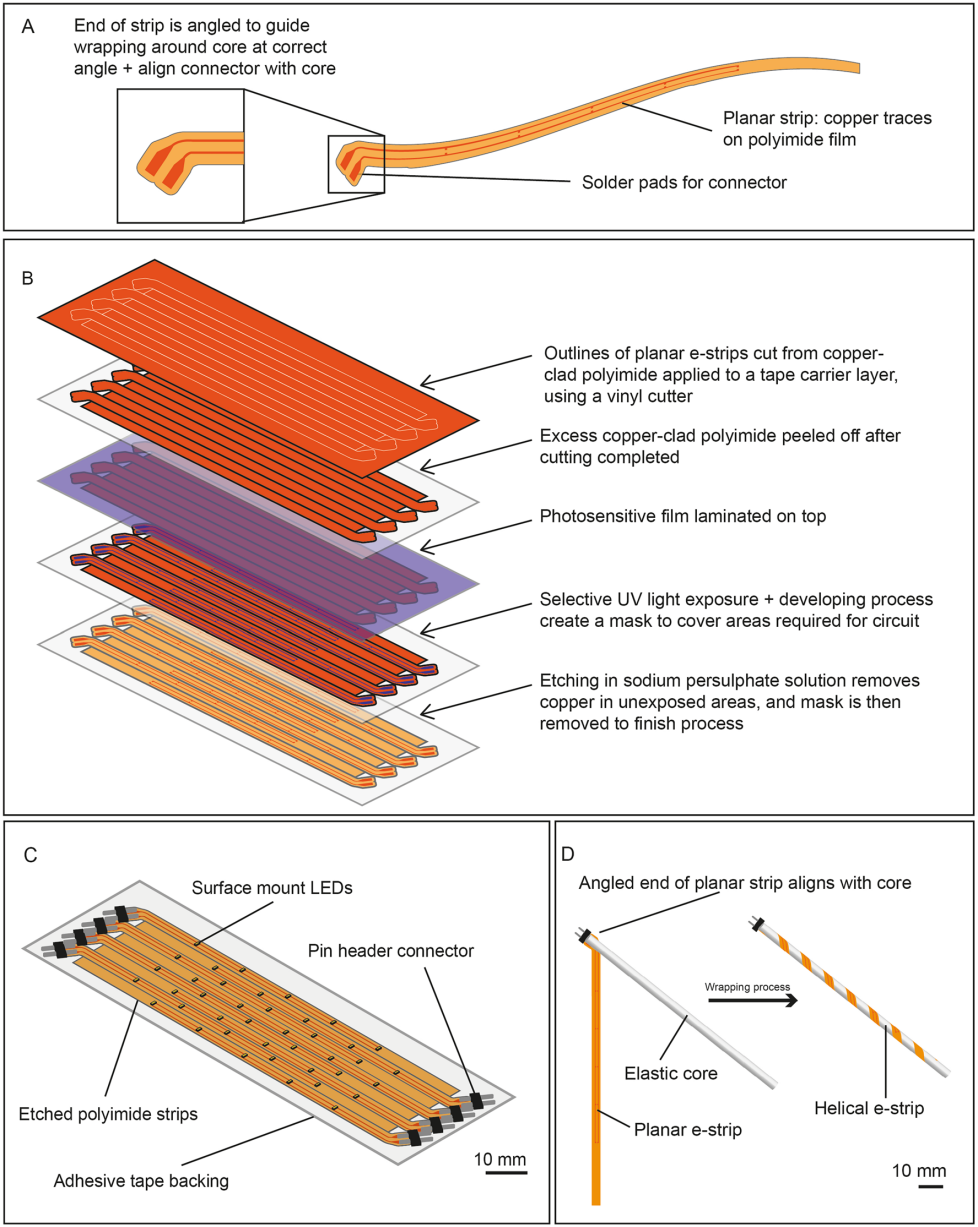
Fabrication process: (**A**) Design of the planar e-strip, with copper traces etched on PI film, and the e-strip end angled so that an attached connector aligns with the core; (**B**) Illustration of the fabrication process of a planar LED e-strip; (**C**) Finished planar e-strips, with components soldered in place and pin header connectors attached; (**D**) Winding process, where the connector end is bonded first, and then the remainder of the planar e-strip is wound around the core and bonded to form the helical geometry.

The fabrication process is illustrated in Fig. 2. A ‘strip’, having length much greater than its width, was fabricated on PI film (“the planar e-strip”). Components were soldered onto the planar e-strip, and adhesive encapsulation was added. Figure 2B shows one end of the e-strip (or both, depending on design) containing solder pads for connectors to be attached, and angled so that the connector is aligned with the core of the helical e-strip. This is matched to the desired helix angle of the helical e-strip. Planar e-strips were fabricated in batches on an adhesive tape carrier, using etching processes to selectively remove copper from copper-plated PI (Fig. 2B,C). E-strips were then wrapped around a rubber core and bonded in place with adhesive, forming the stretchable helical structure in Fig. 2D.

### Optimal geometry and design rules

**Figure 3:**
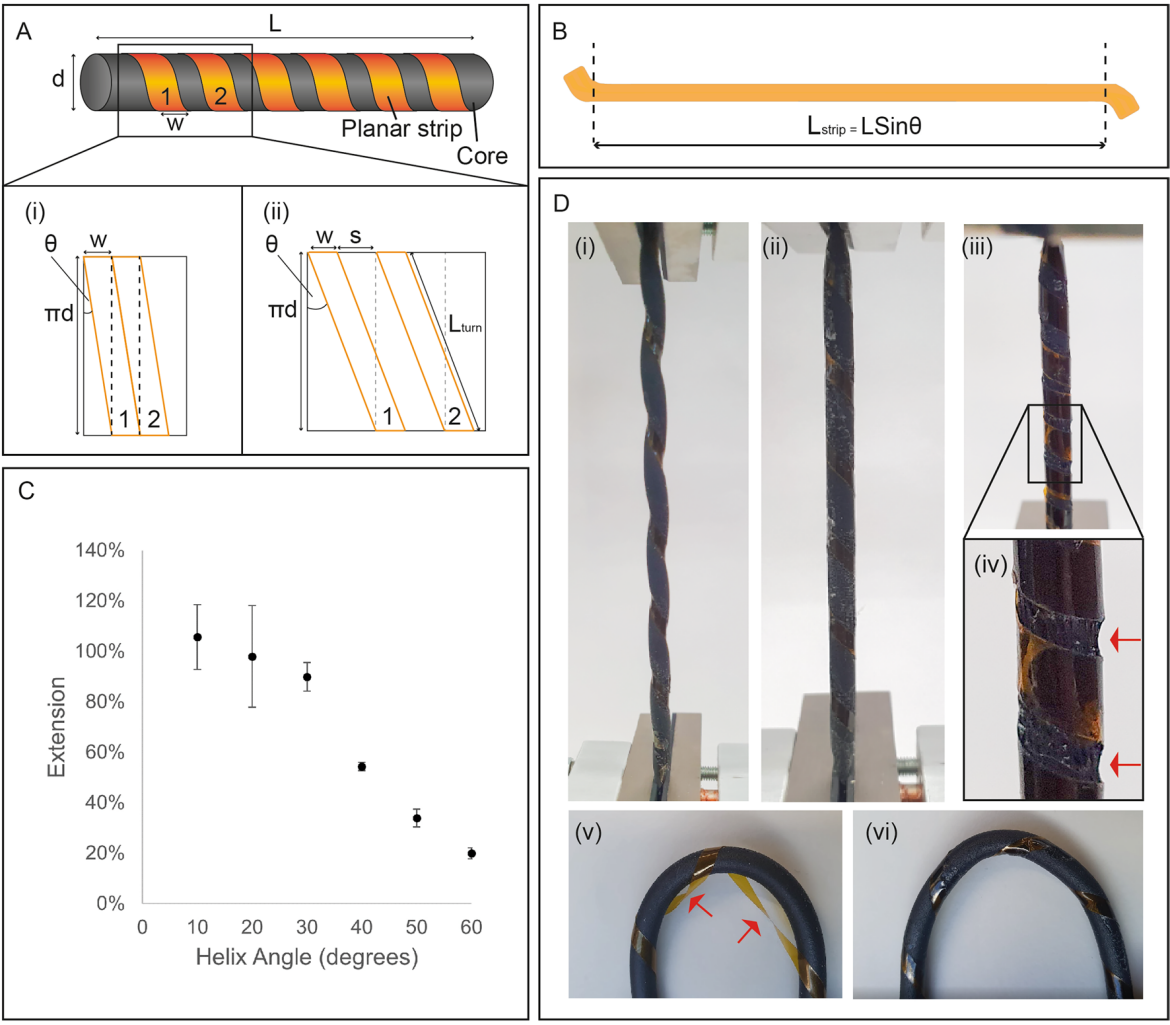
Optimizing geometry: (**A**) (i) simplified illustration showing helix angle, θ, length L, planar e-strip width w; (ii) Minimum helix angle, with no gap between turns of the helix; (iii) General case where a spacing s exists between helix turns, and L_strip_ is the length of planar e-strip required for each turn; (**B**) Illustration of planar e-strip showing length L_strip_ needed to create a helical e-strip of length L; (**C**) Tensile test of blank e-strips of varying helix angle. Here, d = 4 mm and w = 3 mm. 3 samples were tested for each data point, and error bars represent standard deviation; (**D**) Tensile testing images, showing (i) undesirable rippling effect for θ ≥ 40°; (ii) 30° e-strip showing no deformation; (iii) 10° strip during tensile test; (iv) Section of 10° strip showing core damaged occurring at θ < 30°; (v) Image of bent blank strip, showing that helical geometry is lost if the planar e-strip is not bonded to the core; (vi) An identical blank strip but with the planar e-strip bonded to the core, showing that the helical geometry is maintained.

In determining optimal geometry, the aims were to (a) maximize stretch, for compatibility with stretch fabrics; (b) minimize diameter, so that helical e-strips won’t add noticeable, uncomfortable bulk to garments. Consider a simplified helical e-strip of length, *L,* and diameter, *d,* (Fig. 3A). The planar e-strip has width, *w*, and helix angle, *θ*.

If the planar e-strip is wrapped around the core such that successive turns of the helix are immediately adjacent to each other, this defines the minimum helix angle, *θ*_min_, for a given width, *w*. Figure 3B illustrates an unrolled section of the helical e-strip surface, and it follows that *θ*_min_ is given by Eq. 1:

1 $$\theta_{min} = tan^{ - 1} \left( {\frac{w}{\pi d}} \right)$$

Note that increasing *w* also increases *θ*_min_. The upper limit is given by *θ*_max_ < 90°. At 90°, the planar e-strip and core are parallel, and no helical structure can be formed by wrapping one around the other. Prior work has shown that *θ* plays a critical role in helical ribbon stretchability^48^.

As *d* determines how bulky the helical e-strip will be, it should be minimized, and this places constraints on *w*. The size of components required to build a desired circuit, and the complexity of the circuit, i.e., number of traces required to interconnect all components, will likewise affect the width of the planar e-strip. Figure 3C illustrates the general scenario where there is a spacing, *s*, between turns. Helices are normally characterized by pitch (*w* + *s*). But we consider them separately, as two helical e-strips with the same pitch, but different ratios of *w* to *s*, may exhibit different behavior when stretched.

Given *θ*, the length of planar e-strip, *L*_strip_, required to create a helical e-strip of length *L,* is given by Eq. 2. This is useful as it is likely that the desired helical e-strip length will be determined by the design of an e-textile garment, rather than the planar e-strip length being fixed before the garment is designed. That is, a designer would know *L*, and would need to be able to determine the required *L*_strip_.

2 $$L_{strip} = NL_{turn} = \left( {\frac{L}{w + s}} \right)L_{turn} = \left( {\frac{L}{{L_{turn} Sin\theta }}} \right)L_{turn} = LSin\theta$$

*N* is the number of turns of the helix and can be written in terms of *L*, *w,* and *s*, as *w* + *s* is the length of the core covered by each turn of the helix.

The core material also affects helical e-strip stretchability. To investigate this, blank, unpopulated helical e-strips were fabricated: strips of PI film wrapped around and bonded to ethylene propylene diene monomer (EPDM) rubber foam cord. Tensile tests demonstrated that stretchability and helix angle are inversely correlated (Fig. 3C), which aligns with existing work on helical structures. Helix angles above 40° resulted in a rippling effect when stretched (Fig. 3D(i)). As a helix is stretched, it balances this tensile force by untwisting, as well as compressing radially towards its core. This is believed to be the cause of the core rippling deformation, which is an undesirable effect as it may cause rippling of the fabric when embedded in a garment.

At the other extreme, 10° and 20° helix angles exhibited the most stretch, but also showed signs of core damage (Fig. 3D(iii)–(iv)). As the planar e-strip is adhered to the core, sections of the core lying under the planar e-strip are unable to stretch. That is, the sections highlighted by arrows in Fig. 3D(iv) are the only areas free to stretch. As *θ* decreases, the amount of core material uncovered by PI also decreases. Thus, at smaller helix angles, stretching the helical e-strip can cause the core to tear.

A helix angle of 30° showed sufficient stretch without any rippling or damage (Fig. 3D(ii)). 30° was therefore chosen as the optimal helix angle. Further optimization could be possible by testing more angles between 20° and 30°. But for this work covering the initial development of the helical e-strip, these results were sufficient. It is also important to note that these results are specific to helical e-strips with rubber cores, and a smaller helix angle could be used if the helical geometry was formed without a core. This would increase stretchability, but the structure would be unsupported and fragile, and therefore unsuitable for e-textile applications.

Jiang et al. reported an optimal helix angle of 50.69° for printed helical transistors on polyethylene naphthalate (PEN) film wrapped around a PU fiber core^37^. However, their helix angle is defined differently, given by *θ*_Jiang et al. _= 90 – *θ*. Thus, while their FEM simulations and our experimental results are similar, there is a difference of approximately 10°. This may be due to differences in materials. It is expected that optimal helix angle may vary for different core materials or because, in their work, the planar e-strip was wrapped around, but not bonded to the core.

Figures 3D(v)–(vi) show why the planar e-strip was bonded to the core. Without bonding, bending caused the e-strip to lose its helical shape, which could easily cause folding or tearing of the PI film. Bonding ensures that the helical geometry is maintained.

The impact of the ratio of planar e-strip width to helical e-strip diameter was also investigated, and further information is provided in Supplementary Information Section 4. In terms of minimizing diameter, 2 mm was the smallest diameter achieved in this work. In terms of ratio of width to diameter, *w* ≤ *d* is required, and w = 0.75*d* was set as the target for this work. Further information on this is provided in Supplementary Information Section 4. In this work, standard SMD components are used, and the size of these components places constraints on the width of the planar e-strip, and therefore on the diameter. The smallest LED packaged used in this work is 0.6 mm wide, and the planar e-strips made using these LEDs were 1.5 mm wide, to allow additional space for solder pads and clearance between components and the edge of the planar e-strip.

### Helical interconnect e-strips

**Figure 4:**
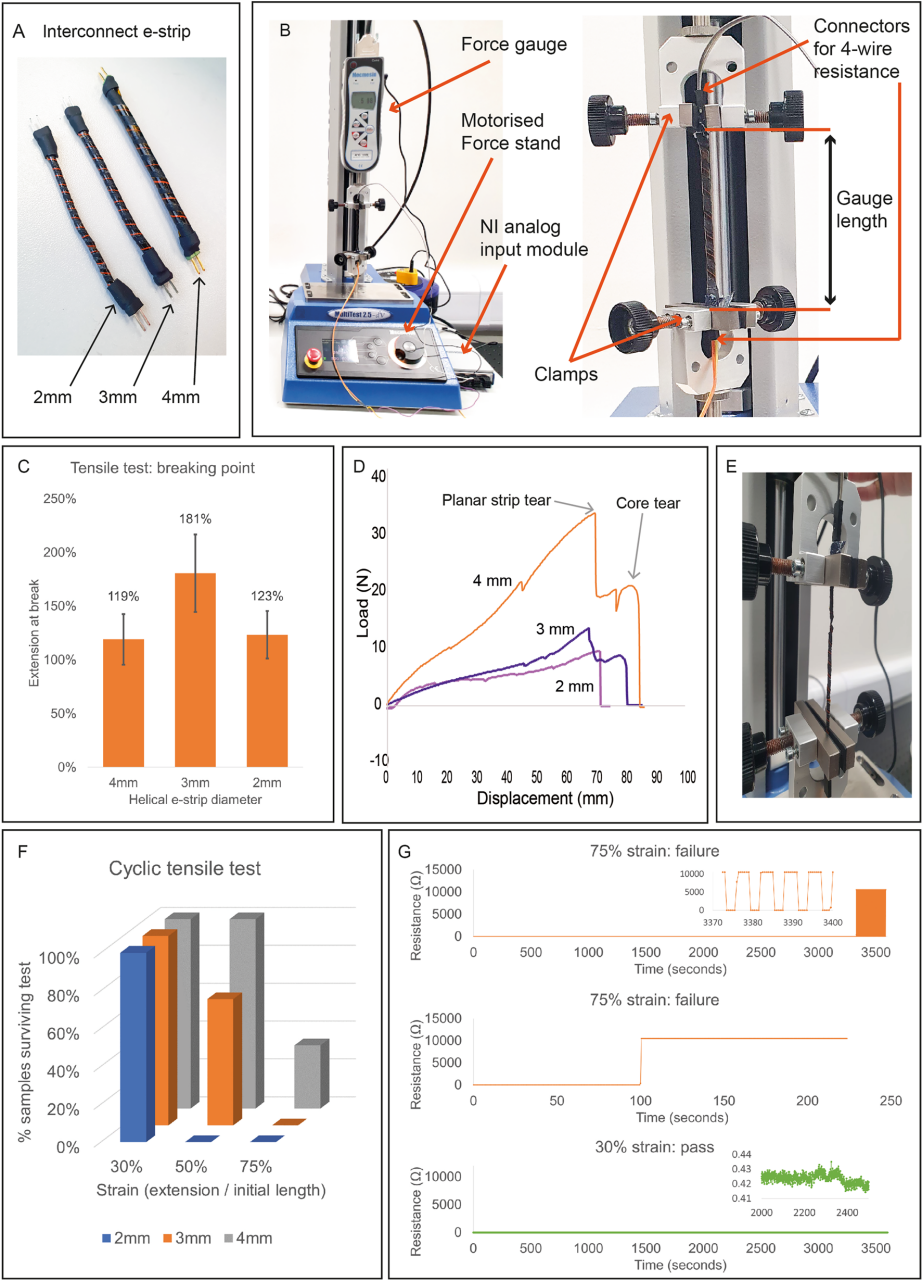
Helical interconnect e-strips: (**A**) Image of 2 mm, 3 mm and 4 mm interconnect e-strips; (**B**) Mechanical test setup for tensile tests; (**C**) results of tensile tests until breaking point. Data points represent the mean of three samples, and error bars represent standard deviation; (**D**) load-extension graph showing stages of failure; (**E**) images of failure modes; (**F**) results of cyclic tensile tests, showing consistent good performance at 30% strain, mixed results at 50% strain, and consistent failure at 75% strain; (**G**) Resistance data from cyclic tests, showing that tearing of the planar e-strip results in a very quick failure, and resistance otherwise remains stable.

Helical interconnect e-strips were fabricated, consisting of one 10 mil (0.254 mm) copper track, with connectors at each end (Fig. 4A). This enabled the four-wire resistance of the helical e-strip to be measured during tensile testing (Fig. 4B). Interconnect e-strips of 2 mm, 3 mm and 4 mm diameter were fabricated, with w = 0.75*d* in all cases. First, three strips of each diameter were stretched until failure. All were able to extend by 100% before failing (Fig. 4C). 3 mm e-strips’ ability to withstand the highest strain was likely because thinner EPDM cords are more elastic, but also more fragile. For 4 mm and 3 mm diameter helical e-strips the planar strip broke first, followed by the core (Fig. 4D). At 2 mm diameter, both tended to break simultaneously, or in the opposite order (Fig. 4E).

To further investigate performance, cyclic tests were performed, whereby e-strips were repeatedly cycled through moderate stretching and relaxing. Three strips of each diameter were stretched by 30%, 50% and 75% for 3000 cycles. All helical e-strips failed the 75% strain test with damage to the core being the most common failure mode (Fig. 4F). At 50% strain, all 4 mm interconnects, and 2 out of 3 3 mm interconnects, passed, but 2 mm interconnects showed signs of core damage. At 30%, interconnects of all diameters passed with no signs of damage. Thus e-strips with 2–4 mm EPDM cores can survive 30% strain, but for garments requiring many cycles of high (> 50%) stretch, a different core material may be required. Overall, helical e-strips tended to remain fully functional until a sudden breakage occurred, rather than degrading slowly over time (Fig. 4G).

### Helical LED e-strips

**Figure 5:**
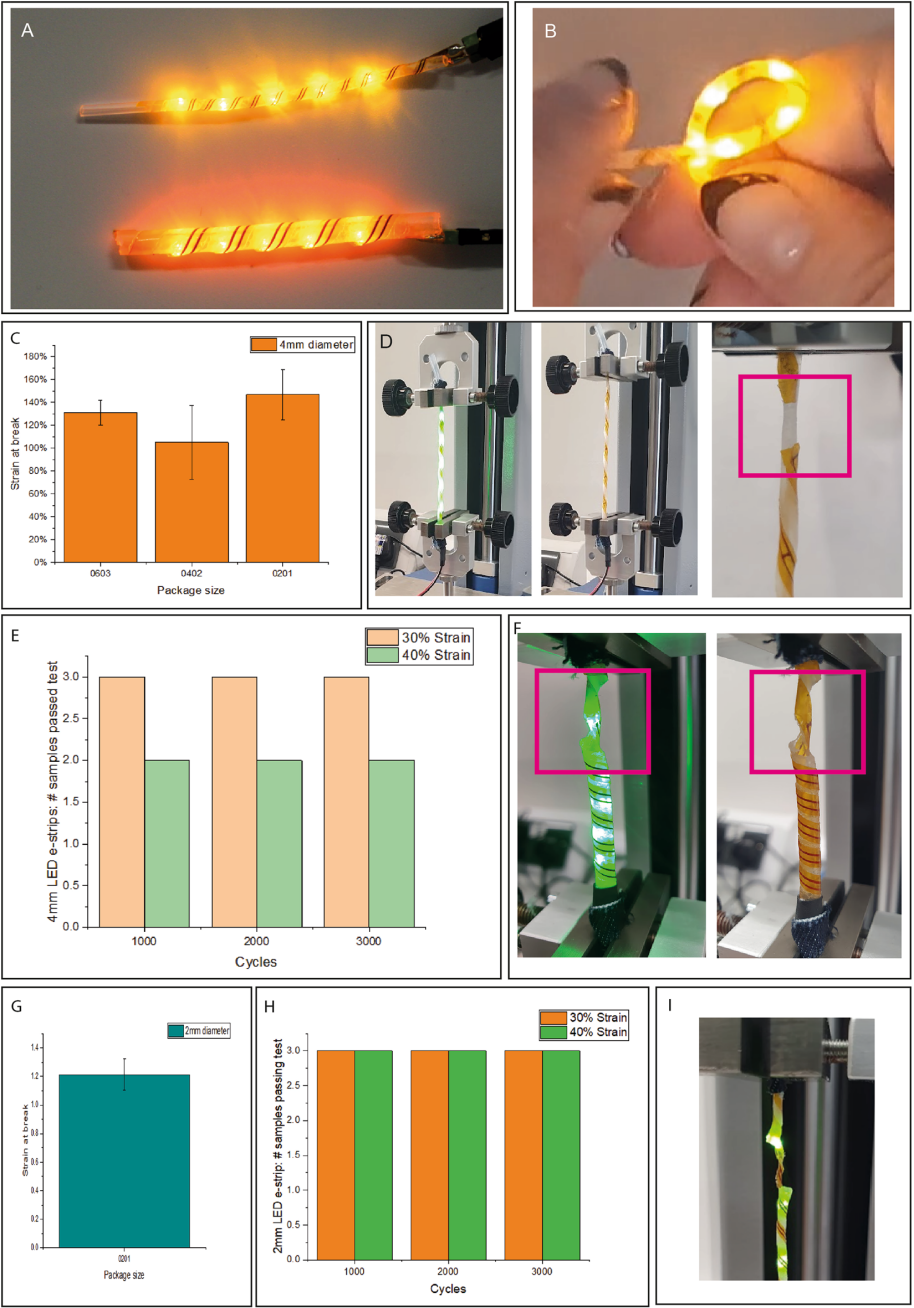
LED-strips: (**A**) Image of 2 mm and 4 mm diameter LED-strips (**B**) planar and helical LED-strips; (**C**) LED-strip tensile tests with different LED package sizes to determine stretchability, where error bars represent standard deviation; (**D**) LED strip under strain, lit (left), unlit due to tear in PI (middle), and a close up of the tear (right); (**E**) Results of cyclic tensile tests of 4 mm diameter LED-strips, showing good results at 30% strain but failures at 40% strain; (**F**) Image of one type of failure mode, where the core has torn but the LED circuit is still functional; (**G**) tensile test result for 2 mm diameter LED-strips; (**H**) Cyclic tensile test results for 2 mm LED-strips, showing improved performance relative to 4mm; (**I**) close-up of 2 mm LED-strip failure, where the core has torn.

Helical LED e-strips (“LED-strips”) were fabricated, consisting of 5 LEDs in parallel (Fig. 5A,B). This demonstrates the potential for helical e-strips to be formed of functional circuits containing components, and could find use in commercial sportswear as an indicator of a sensor recording temperature or heart rate, or for safety purposes including illumination during night-time sporting activities.

As components on helical e-strips face the core, a transparent core was required. Clear silicone tubing was chosen as it is transparent, but also compressible, allowing components to press into the core and avoid creating an uneven outer surface. The orientation of the components further allowed light to diffuse through the core (Fig. 5A).

LED packages used were: 0603 (1.6 mm × 0.8 mm), 0402 (1.0 × 0.5 mm) and 0201 (0.6 mm × 0.3 mm), the smallest standard commercially available sizes. Smaller LEDs are not readily available from electronics suppliers in the UK, where this work was performed, and not yet widely used in electronics manufacturing due to their small size^49^. LED package sizes are given in imperial units, and all LEDs in this work were 0.2 mm tall.

#### Tensile testing

4 mm diameter LED-strips, with 0603, 0402 and 0201 LEDs, were stretched until failure, i.e., until one of more of the LEDs turned off, indicating a crack or tear (Fig. 5C). Two failure modes were observed (Fig. 5D): (a) the PI film tore near one of the clamps, causing all LEDs to turn off, (b) the planar strip detached from the core, a failure mode not observed with interconnect e-strips as EPDM is easier to bond than silicone. Although 0201 LED-strips were the most stretchable (breaking at ~ 140% extension), there was high variation between samples, and it was not clear that package size significantly impacted stretchability. The main takeaway was that all LED-strips could withstand > 100% strain.

Cyclic tensile tests were also performed on 4 mm LED-strips, and samples survived 3000 cycles of 30% stretch with no signs of physical or electrical damage (Fig. 5E). Increasing strain to 40% resulted in physical damage to the core of the LED-strips (Fig. 5F), LEDs were still functional after the test. Therefore 4 mm LED-strips are suitable for integration into moderate (20–30%) stretch textiles, but a more elastic core would be required for fabrics with higher stretch.

After validating the concept with 4 mm diameter LED-strips, 2 mm diameter LED-strips were fabricated, using 2 mm diameter silicone tubing and 0201 LEDs. These are more suitable for integration into textiles, with their smaller diameter making them less noticeable in the garment. Subjected to the same tests, 2 mm LED-strips broke at approx. 120% strain (Fig. 5G), not significantly different to the 4 mm LED-strips. However, 2 mm LED-strips demonstrated better performance in cyclic tensile tests (Fig. 5H), with all surviving not only 3000 cycles of 30% strain, but also 40% strain. This is attributed to the core being more elastic: both are silicone tubing, but the thinner 2 mm cords, are more stretchable. Figure 5I shows an example failure mode of 2 mm LED-strips.

### Wash testing

**Figure 6:**
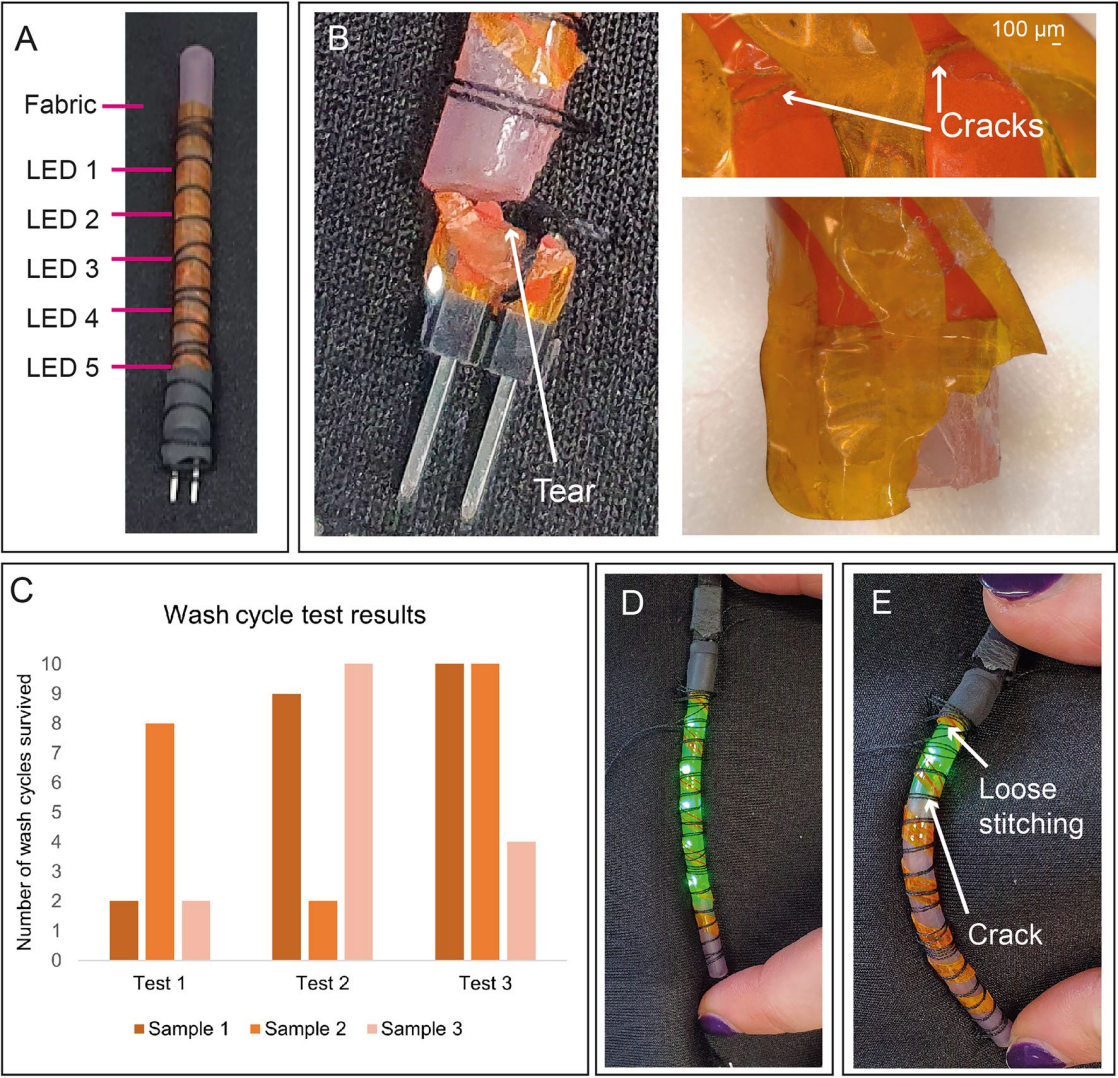
Wash testing of LED helical e-strips: (**A**) LED-strips stitched onto a piece of fabric for washing; (**B**) Image and microscope image of tear adjacent to connector in sample from first wash test; (**C**) Wash test results, showing that more samples survived washing when area adjacent to connector was reinforced with heat shrink (Tests 2 and 3), and some samples survived 10 wash cycles; (**D**) Failure mode observed in third wash test, where a crack has formed in the conductive track, and LEDs turn off when the strip is flexed, opening the crack.

E-textiles need to be washable. Wash tests were therefore performed, using 4 mm diameter LED-strips. Test samples consisted of 3 LED-strips stitched onto a fabric piece (Fig. 6A). These were placed in a washing machine and washed on a 40 °C/1400 rpm cycle with liquid detergent. According to a study on e-textile wash testing^50^, this is similar to many other e-textile wash tests in terms of temperature and the use of liquid detergent. However, spin cycle settings in wash tests are rarely reported. As domestic washing machines typically advertise spin speeds of between 400–1800 rpm, a high speed was chosen to subject the helical e-strips to maximal stress and identify failure modes quickly. A gentler cycle designed for delicate fabrics could extend the washing durability, but subjecting helical e-strips to high stress to identify failure modes was prioritized at this stage.

The first test used LED-strips without heat shrink encapsulating the interface between connector and strip (Fig. 6B). As seen in Fig. 6C (Test 1) two of the e-strips in this sample failed on the third wash cycle, due to tearing of the planar e-strip next to the connector. The third survived 9 washes before suffering the same failure. After the final test, probing with a multimeter confirmed that the tear was the only form of damage, and all LEDs on the broken e-strips still functioned.

Two further samples were created, using heat shrink, one with 0603 LEDs and one with 0402 LEDs. Results are shown in Fig. 6C (Tests 2 and 3, respectively). Stitching loosened in one sample, causing a tear near the connector. Some other LEDs came loose, but most samples survived 10 washes. A semi-functional sample is shown in Fig. 6D and E. A crack broke the circuit when the strip was bent, but the LEDs still lit when released. Overall, further work is required to produce more robust helical e-strips, but these results are promising.

### Helical sensing e-strips

**Figure 7:**
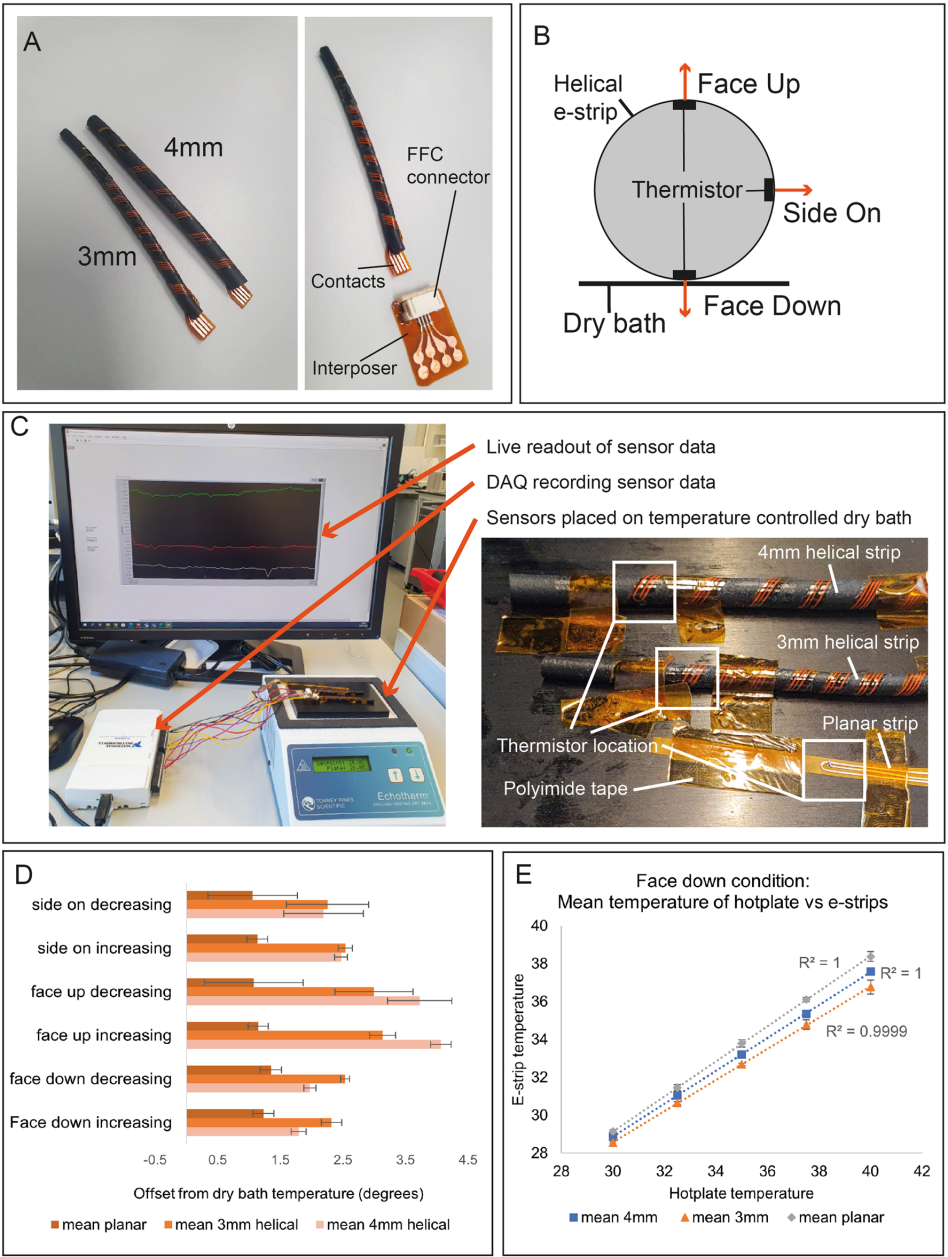
Temperature sensing helical e-strips: (**A**) Image of the temp-strip with 4 mm and 3 mm diameter, and interposer for interfacing with measurement equipment; (**B**) Illustration of experimental conditions, where the thermistor is oriented directly in contact with the heated surface (Face Down), at 90° away from the heated surface (Side On), or 180° away (Face Up); (**C**) Experimental setup of dry bath, temperature sensing helical e-strips, and close-up of sensing e-strips on dry bath surface, with thermistor location highlighted; (**D**) Results showing performance of temp-strips relative to planar e-strip during different conditions; (**E**) Performance of e-strips in Face Down condition across temperature range, showing linear offset in temperature. Data points in graphs represent mean values averaged over 2 min of recorded data, and error bars represent standard deviation.

Temperature sensing helical e-strips (“temp-strips”) were fabricated to demonstrate helical e-strip applications in physiological sensing (Fig. 7A). A flexible temperature sensor was adapted from previous work^51^, using 10 kΩ NTC thermistors, as these have good accuracy within human body temperature range, and have been used in existing work^52^.

For skin temperature measurement, good contact between sensor and skin is key for accurate results^53^. Other studies used planar electronics where the orientation of the temperature sensor is fixed^18^, or where a thermistor embedded in a yarn may rotate, but has symmetric structure so that rotation did not impact the distance between thermistor and skin^54^. In the helical e-strip, it is expected that orientation is important, as components are not in the center of the helical e-strip, and should the e-strip rotate, the position of the thermistor relative to the skin may change. As this may affect results, a characterization experiment was performed.

As skin temperature is normally higher than ambient temperature, it is important to replicate these conditions during tests. The orientations of the temp-strips were varied (Fig. 7B), with three conditions: ‘Face Down’, ‘Face Up’, and ‘Side On’. A planar e-strip, a 3 mm temp-strip, and a 4 mm temp-strip were attached to a hotplate (Fig. 7C). Each test was run twice, once with the temperature decreasing from 40° to 30°, and then increasing from 30° to 40°. Best results were obtained in the Face Down condition (Fig. 7D). The 4 mm temp-strip obtained more accurate results than the 3 mm temp-strip, which may be because the 4 mm core provides more insulation between the thermistor and the environment, preventing thermistor readings from drifting towards ambient temperature^55^.

Helical e-strips were slower to respond to changes in temperature, which is attributed to the core material retaining heat (Supplementary Information Section 6). In all cases the error was linear (Fig. 7E) and could be removed by calibrating the sensor.

From this we can conclude that in a real application it would be important to fix the part with the sensing element so that it is facing the interior of the garment, to prevent rotation and ensure accuracy. For other types of sensors, i.e., motion sensing using accelerometers, contact with the skin is not important, so this would be less of a concern. But as embedding sensors in textiles is known to affect their performance^52^, additional testing would be required to calibrate temp-strips for use in a garment.

## Discussion

The potential of 3D helical structure for embedded electronics in e-textiles, prioritizing standard components and materials, has been investigated and supported with experimental evidence. A helical e-strip fabrication process has been developed, defining design rules, fabricating prototypes, and performing mechanical testing to validate the concept, and identify areas for improvement in future iterations. Functionality has been demonstrated through helical interconnect, LED and temperature sensing e-strips. Circuits with 1, 2 and 4 tracks have been successfully fabricated, using 0201, 0402 and 0603 SMD components. Helical interconnect e-strips with 4 mm diameter can withstand 50% strain for 3000 cycles, and 4 mm LED-strips, having a slightly less stretchable core, can survive 3000 cycles of 30% strain, and up to 10 wash cycles. 2 mm LED-strips were undamaged after 3000 cycles of 40% strain. Longer tensile tests, and additional mechanical tests, were not performed at this stage.

As e-textile standards are still in development, tests to evaluate e-textiles vary significantly. The key questions are: (a) how much do e-textile parts need to stretch, and (b) how many stretch cycles should they survive? The first question depends heavily on the fabrics used. Different textiles, made with different fibers, using different techniques, vary significantly. While some materials such as spandex can stretch more than 100%, spandex garments also contain seams, which are less stretchy, and zippers, which do not stretch at all. Therefore, e-textile circuitry doesn’t necessarily need to be as stretchable as spandex, and 30% stretch may be sufficient for many applications. The second question relates to durability, and how a garment is to be used: is it a garment where circuitry will be routed along the elbows or knees, and which will be subjected to a lot of bending and stretching? Or is it a compression garment covering only the forearm, which will be pulled on and then remain at more or less constant elongation while worn?

Helical e-strips can achieve 100–180% elongation before breaking (depending on core material and diameter). This is higher than the 79–88% range achieved with serpentine-shaped PI interconnects encapsulated in TPU, in work aimed at a similar application^18^. Serpentine tracks on elastomer substrates have demonstrated 260% stretch, but without cyclic testing to assess durability^19^. Bossuyt et al. suggest that an e-textile sensor with an intended lifetime of a year or longer should survive 10^4^ cycles of 5–10% stretch, and 10^6^ cycles of 1–3%^22^. But this depends on the application, and there is a lack of data quantifying the mechanical strain e-textile parts experience in a garment. 30% strain, for 1000 cycles, has been used in similar work^18,56^. And highly stretchable helical interconnects have survived 500% strain, but only for 500 cycles^33^. Thus helical e-strips, at this early development stage, perform well against competing technologies.

The main failure modes observed were (a) planar e-strip tearing at the interface between connector and helix; (b) core material tearing; (c) delamination of planar e-strip the core, or delamination of components from planar e-strip. All can be improved by (a) applying strain relief; (b) investigating alternative adhesives, and (c) applying additional stretchable encapsulation to the exterior of the e-strips. Overall, the viability of the concept has been validated, and future work will improve reliability by refining the fabrication process and materials.

Prototyping and initial development of the helical e-strip have been covered by this work, but moving forward to manufacturing, a key area for development is sustainability. Cyanoacrylate adhesive was used to bind the planar e-strips to the core, but this is difficult to remove for repair, or recycling. However, an advantage of helical e-strips is that they don’t rely on specific materials. The structure could be constructed from printed circuitry on a different substrate, or using a different core material or adhesives. Future developments in electronics—thinner, more flexible components—will also benefit helical e-strips, rather than making them redundant.

This work has demonstrated helical e-strips with one layer of circuitry. Future work will focus on multi-layer structures to realize more complex circuits. And minimizing diameter, for more seamless integration into textiles, as component size is a key limiting factor on helical e-strip diameter. As mentioned in Sect. 2, the narrowest helical e-strips fabricated in this work were 2 mm in diameter, with a 1.5 mm wide planar e-strip. As many textiles are made of threads and yarns less than 1 mm in diameter, future work could investigate the use of printed or bare die components, or new technologies such as flexible ICs^57^, or thermally drawn digital fibers^58,59^. This would enable the diameter of helical e-strips to be reduced, and facilitate integration into thin fabrics without adding noticeable bulk. Other areas of potential future work include investigating different core materials, such as silicones with lower shore hardness, or PDMS. And manufacturing, including increasing the sustainability of the manufacturing process, and developing an automated process to create the helical geometry, for example by adapting textile braiding technology, which has been used in e-textiles and similar applications^36,60^. In summary, this work has explored, and validated, the viability of creating circuits in a helical geometry, which has the potential to transform stretchable electronics technology for e-textiles.

## Methods

### Design and fabrication

#### Choice of planar e-strip substrate

Copper-clad PI film was chosen for the planar e-strip, due to its widespread use in flexible electronics and compatibility with existing manufacturing processes, i.e., its high melting temperature means it doesn’t deform during soldering. To form circuit traces, etching copper-clad PI film was chosen over alternative methods to fabricate flexible electronics, e.g., screen printing or direct ink write printing on PI or other flexible substrates. Printing cannot produce very fine pitch features, so etching fabrication processes were chosen so that circuits using components and conductive tracks with < 1 mm pitch could be fabricated. A clear flexible substrate such as PEN film could also have been used for LED e-strips to maximize transmitted light, but as the focus of this work was on proving the concept of the helical e-strip, the more easily available PI was used.

Three variants of copper-clad PI film (GTS Flexible Materials, Ebbw Vale, UK) with varying thickness of the PI layer were tested: 25 µm, 50 µm and 75 µm. The 25 µm film (copper thickness: 18 µm in all cases) was selected for use in the helical e-strips, as it is thick enough to support SMD components but thin enough to wrap easily around rubber foam cores down to 2 mm in diameter. 25 µm PI film with no copper coating was used to form ‘blank’ strips fabricated to investigate fundamental helical e-strip geometry.

#### Selection of the core material

Rubber cord was chosen for the core material as it is stretchable, compressible, and widely available. For interconnect and temperature sensing strips, rubber foam cord was tested, as the foam structure makes it more compressible than other rubber cords, and this is beneficial as components face the interior of the helical e-strip and must compress into the core material. Silicone, EPDM, and neoprene foam cords (Polymax Ltd, Southampton, UK) were selected as potential core materials, as all are waterproof and are widely available. All three were subjected to tensile and compression testing based on ISO 20932-1:2020 + A1:2021^61^ and ISO 7743:2011^62^, to compare their stretchability, compressibility and ability to recover after tensile testing. EPDM was ultimately selected, as it performed the best across all categories. Detailed results are included in Supplementary Information Section 2. Only commercially available cords were used, instead of casting custom cords, to ensure consistent high quality, and no irregularities in the core structure.

**Figure 8:**
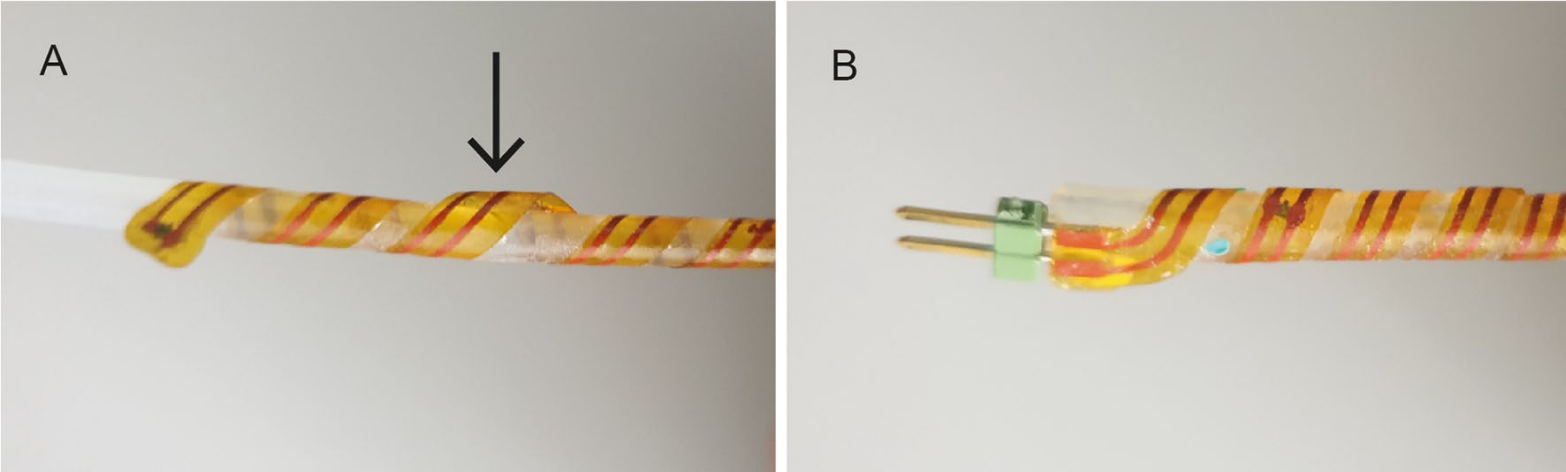
Evaluation of core materials for LED helical e-strips: (**A**) Solid silicone cord: arrow highlights an area where the inability of components to compress into the core can be seen, resulting in an uneven surface; (**B**) Silicone tubing core: the surface of this e-strip is much smoother as the higher compressibility of the silicone cord allows components to compress the surface.

For helical LED-strips, a clear core material was needed to transmit the light. Translucent silicone cord and silicone tubing both with a shore hardness of 60 were both evaluated. As can be seen in Fig. 8A, the silicone cord was not compressible enough, preventing components from embedding into the core surface, and resulting in an uneven structure. Silicone tubing, depicted in Fig. 8B, resulted in a much smoother surface, and was thus chosen as the LED helical e-strip core material.

#### Components

As discussed in Sect. 2, component footprint has an impact on the minimum strip diameter. Helical e-strips were first constructed with 0603 (metric 1608) SMD components, to validate the concept. Further prototypes were fabricated with 0402, and 0201 package components, the smallest standard sizes used in electronics manufacturing, to enable smaller diameter helical e-strips to be made. Temp-strips were made with 0201 components only, formed in a Wheatstone Bridge configuration.

2.54 mm pin headers were used as connectors for interconnect and LED e-strips, to enable easy connection to power supplies and DAQ devices for testing. In a real garment a latching mechanism would also be needed to prevent disconnection. For sensing e-strips, which required a 4-pin connector, an interposer containing a smaller footprint FFC connector (Molex Ltd, Illinois, USA) was fabricated. For the temperature sensing circuit, a Wheatstone bridge was constructed using a10 kΩ NTC thermistor and three 10 kΩ resistors (RS Components Ltd, Corby, UK). Both interposer and temperature sensing circuit are based on previous work^63^. Components were soldered to planar e-strips using T5 solder paste (Chipquik Ltd, Ontario, Canada) and a hotplate.

#### Bonding and encapsulation

Adhesives were used for a) encapsulating components and copper traces, to reduce the likelihood of them breaking off the PI substrate, and b) bonding the planar e-strip to the core. For component encapsulation, flexible medical adhesive (Dymax Ltd, Torrington, CT, USA) was dispensed onto the planar e-strips. A syringe was used to dispense adhesive on top of components as glob top encapsulation, and a brush was used to spread a thin layer of adhesive onto the remainder of the e-strip, to insulate the copper tracks. To bond planar e-strips to the core, several adhesives were tested: silicone adhesive (Wacker Chemie AG, Munich, Germany), flexible cyanoacrylate (Intertronics, Kidlington, Oxfordshire, UK), and bonding tapes for flexible electronics (3 M, Minnesota, USA). These were assessed both on ease of use in the fabrication process, and their ability to hold the e-strip together when stretched.

FPC tape was found to be unsuitable for bonding the planar e-strip to the core, as due to the helical structure of the e-strip, and the compressible core, it was not possible to apply pressure to ensure proper bonding of the tape. Silicone adhesive was also ruled out, as although this created a flexible bond, its 24-h curing time resulted in some e-strips untwisting after initial bonding, and for this work a quick curing adhesive was needed. The flexible cyanoacrylate adhesive was chosen as the preferred bonding method, as its strong bond and fast curing time formed a very secure structure. For LED e-strips, which used a silicone core, an additional cyanoacrylate primer was required for successful bonding.

Heat shrink was also used as a kind of encapsulation or strain relief on the interface between connectors and the body of the e-strip. Boundaries between rigid and flexible materials in electronics are known to be susceptible to failures, so the addition of heat shrink aimed to reduce the mechanical strain in this area when the helical e-strips were under strain.

### Fabrication

The fabrication process has three key stages, which are:

1. Fabrication of the planar e-strip: Etching copper-plated PI film, soldering SMD components onto the e-strip if required, soldering connectors, and applying encapsulation.
2. Forming the helical geometry: Winding the planar e-strip around the core and bonding it with flexible cyanoacrylate adhesive
3. Additional encapsulation: Applying heat shrink to the interface between the connector and the stretchable part of the helical e-strip, to add mechanical support to this area.

A detailed step-by-step process is included in Supplementary Information section Section 4.

### Evaluation

#### Testing methodology

##### Tensile testing

Tensile tests were based on IPC-9204 testing methods for flexible electronics^64^, and performed with a universal testing machine (Multitest 2.5 dV model, Mecmesin/PPT Group UK, Horsham, UK) equipped with a 100 N force gauge and rubber-coated grips. Samples were clamped at each end, and the gauge length was measured with a steel ruler. Mecmesin VectorPro Lite software (Version 7.0.0.0 https://www.mecmesin.com/software/vectorpro-lite-software-force-instruments) was used to program tests, and record load and extension data.

Blank e-strips were extended at a rate of 50 mm/min until a load of 10 N was applied, or until they broke, if that happened sooner. For the remainder of the tensile tests, small strips of fabric were glued on top of the heat shrink on e-strips undergoing tensile testing, to improve grip and prevent the e-strips from slipping out of the universal testing machine’s clamps. Mechanical testing of interconnects also included continuous measurement of 4-wire resistance to detect damage to copper tracks. This was measured using an NI-9219 analog input module (National Instruments Ltd, Texas, USA), connected via USB to a laptop where data was recorded using NI LabVIEW software (version 2019, https://www.ni.com/en/shop/labview.html). Cyclic tests were conducted at a rate of 800 mm/min.

Tested samples were also assessed by visual inspection, i.e., determining whether a sample had passed or failed a test based on signs of physical damage. LED-strips were connected to a bench power supply and stretched while lit, so that damage was indicated by one or more LEDs flickering, dimming, or turning off. A Keyence VHX-7000 microscope was also used for more detailed inspection of failures, such as cracks formed in copper tracks or breaking of solder joints.

##### Wash testing

A domestic washing machine (Model i-DOS, Bosch GMBH, Gerlingen, Germany) was used to perform wash tests. A generic supermarket brand non-bio detergent was added to the washing machine, which uses intelligent dosing to determine the appropriate amount of detergent for each cycle. An additional load ballast of 2.2 kg polycotton fabric was added, in accordance with ISO 6330 washing standard^65^, which was used as a guideline for wash tests. Samples were washed in a 40 °C, 1400 rpm cycle lasting 2 h 33 min, to replicate a regular domestic wash cycle. Samples were air dried in between washes, and weighed on a balance before each wash to ensure that they were fully dry and had not retained any moisture, before the next wash cycle began.

##### Temperature sensor characterization

Sensing e-strips were characterized using a digital chilling/heating dry bath (Torrey-Pines Scientific Inc, Carlsbad, CA, USA). 4 mm and 3 mm diameter helical e-strips, and a planar e-strip, were attached to the surface of the dry bath using PI tape, taking care not to place tape over the section of the e-strips where the thermistor is located. Each e-strip was connected to an interposer: a flexible circuit module routing the e-strip contacts to wires, enabling connection to screw wire terminals on a DAQ device (NI-USB-6210, National Instruments Ltd, Texas, USA) which transmitted data to a computer. LabVIEW software (same version as above) was used to record sensor voltages and calculate and record temperature values from these.

During the tests, the planar e-strip was placed flat on the dry bath surface. The helical e-strips were placed such that the thermistor was facing down onto the dry bath surface (Face Down condition), at 90° (Side On condition) or on the opposite side (Face Up condition), as illustrated in Fig. 6B. For each test, the hotplate temperature was increased from 30 to 40 °C in steps of 2.5 °C. This range was chosen as it covers the normal range of human skin, and this technology is intended for integration into e-textile garments. After each temperature increment, the sensor readings were allowed to stabilize for 2 min, and then data was collected for a further 2 min. Data was collected a rate of 2 Hz.

### Supplementary Information

Supplementary Information 1.

Supplementary Video 1.

## Data Availability

Data supporting the results presented in this paper are available from the authors on reasonable request to the corresponding author.
