# Supplementary material for: Stretchable electronic strips for electronic textiles enabled by 3D helical structure
Source: Sci Rep. 2024 May 14;14:11065. doi: 10.1038/s41598-024-61406-7 (PMC11094078; doi:10.1038/s41598-024-61406-7)
Supplement: Supplementary file 1 — Supplementary Information 1. [file 41598_2024_61406_MOESM1_ESM.pdf]

Stretchable electronic strips for electronic textiles enabled by 3D helical structure

Jessica Stanley[1,2], Phil Kunovski[3], John Hunt[2,4], Yang Wei[1,2]

[1] Smart Wearable Research Group, Department of Engineering, Nottingham Trent University, UK

[2] Medical Technologies Innovation Facility, Nottingham Trent University, UK

[3] Kymira Ltd, Reading, UK

[4] College of Biomedical Engineering, China Medical University, Taichung 40402, Taiwan

Supplementary Information

1 Additional images of helical sensing e-strip in fabric

Supplementary Figure 1 shows additional images of the temperature sensing helical e-strip embedded in a concealed channel in an armband (made grey marl terry fabric, acquired from Hobbycraft Ltd, UK). A strip of the fabric was used to create a concealed channel in the armband, by stitching it onto the fabric with a zigzag stitch to allow the channel to stretch.

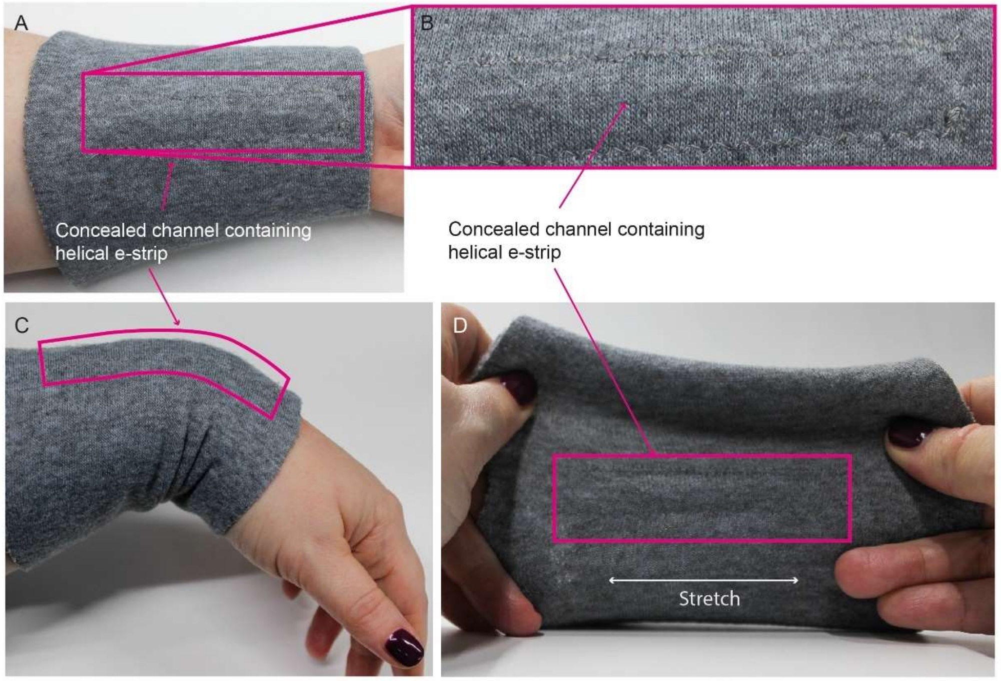

Supplementary Figure 1. Additional images of helical temperature sensing e-strip embedded in a concealed channel in fabric: A) Fabric armband containing helical e-strip, with location of channel highlighted; B) Close-up image showing that the location of the e-strip is barely visible; C) Bending the wrist to show that the helical e-strip bends along with the fabric; D) Stretching the fabric.

2 Helical e-strip fabrication

Four categories of helical e-strip were constructed, as detailed in Supplementary Table 1.

Supplementary Table 1: Categories of helical e-strips fabricated in this work, with their materials and characteristics

E-strip category	Materials (excludes adhesives + solder)	Diameters (mm)	Helix angles (degrees)	Planar e-strip widths (mm)
Blank	Polyimide film, EPDM core	2, 3, 4, 5	10, 20, 30, 40, 50, 60	2, 3, 4, 5
Interconnect	Copper-clad polyimide film, EPDM core, pin header connector	2, 3, 4	30	1.5, 2.25, 3, 3.75
LED	Copper-clad polyimide film, EPDM core, pin header connector, SMD LEDs	2, 4	30	1.5, 3.5
Sensing	Copper-clad polyimide film, EPDM core, SMD thermistor and resistors	3, 4	30	3

Fabrication of helical e-strips was performed using a variation on a standard PCB fabrication process, as follows:

1. The circuit layout was created in Autodesk EAGLE and edited in Adobe Illustrator to create outlines for the planar e-strip.
2. Using a vinyl cutter (Model GX-24, Roland DGG, Hamamatsu, Japan), the planar e-strip outlines were cut from copper-clad polyimide, mounted on a transfer tape backing to preserve alignment. Excess material was peeled off, as shown in Supplementary Figure 2A.
3. A mask was fabricated to allow the circuit to be etched on the planar e-strip outline.
    a. For helical interconnect and 4 mm diameter LED e-strips, a dry film photolithography process was used. This involved:
        i. Using a vinyl cutter to create a negative UV mask from black heat transfer vinyl. Heat transfer vinyl is a polymer film with a heat-activated adhesive backing, designed for making graphics on clothing. However, as it comes on a clear backing film, it can also be used to make a mask for photolithography. Black Cricut Sportflex heat transfer vinyl was cut to create masks, as shown in Supplementary Figure 2-B. This method was found to be preferable over the more standard method of printing masks on transparent film using an inkjet printer, as the printer used was not able to print the mask so that it was completely opaque.
        ii. Applying photosensitive dry film to the planar e-strip outlines using a laminator
        iii. Placing the UV mask on top of the planar e-strip outlines and taping it in place
        iv. Exposing the photosensitive film to 30 s of UV light to crosslink and harden exposed areas of the film, using a UV exposure box

v. Removing the UV mask, and using a potassium carbonate developer solution to wash away unexposed dry film. The developer solution was constantly agitated by an orbital shaker to aid the developing process.

b. For temperature sensing e-strips and 2 mm diameter LED-strips, the dry film process couldn't be fine-tuned sufficiently to achieve the resolution for these fine pitch circuits. To overcome this issue, an alternative method was developed. Adhesive vinyl, which is similar to heat transfer vinyl, except that its adhesive doesn't need to be heat activated, was applied on top of the planar e-strip outlines before vinyl cutting. Adjusting the cutting force of the vinyl cutter allowed the adhesive vinyl to be cut to form a mask for etching. Excess vinyl was removed with tweezers. This is shown in Supplementary Figure 2-C.

4. Etching in a sodium persulphate solution in a bubble etch tank removed excess copper.
5. Removal of the dry film mask was performed by soaking in acetone, and the vinyl mask was removed using tweezers.
6. Solder paste was dispensed onto solder pads, and SMD components were placed using a pick and place machine.
7. Components were soldered using a hotplate, with the exception of pin header connecters, which were hand soldered using a soldering iron.
8. Encapsulation was applied and exposed to UV light to cure in accordance with manufacturers' recommendations.
9. The planar e-strip was bonded to the core using cyanoacrylate adhesive. First, one end of the planar strip was bonded to the core. Then, using a printed guide to maintain correct helix angle, and silicone paper to prevent any accidental bonding between the helical e-strip and the surface on which it was assembled, the planar e-strip was gradually wrapped around the core and bonded. This process is shown in Supplementary Figure 2-D.
10. Heat shrink was applied to the interface between connector and the rest of the e-strip, and activated by a hot air gun to make it conform tightly to the e-strip. Small amounts of cyanoacrylate were applied between heat shrink and e-strip using a syringe, to make sure it stayed in place during tensile testing.

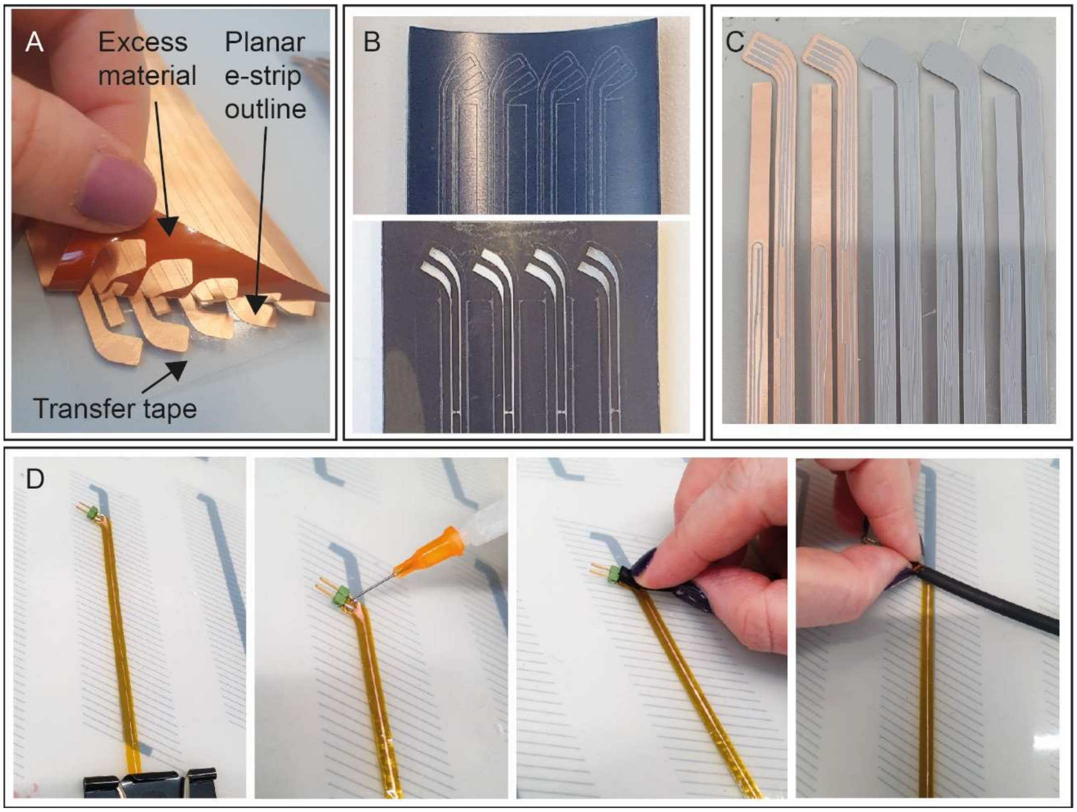

Supplementary Figure 2. Fabrication of the helical e-strip: A) Excess material is removed from planar e-strip outlines after the outlines are cut with a vinyl cutter; B) UV mask for photolithography fabricated from heat transfer vinyl, after vinyl cutting the circuit pattern (top) and after removing the excess vinyl with tweezers (bottom); C) Adhesive vinyl mask applied to temperature sensing e-strip outlines; D) Forming the helical structure: the planar e-strip is placed on silicone paper with a printed grid underneath, then cyanoacrylate adhesive is applied, and the core is adhered to the end of the e-strip. The planar e-strip is then rolled along the core, adding further adhesive (not pictured) to form the helical structure.

3 Evaluation of materials for the helical e-strip core

The material used as the helical e-strip core needed to satisfy the following requirements:

1. Stretch: the core must be highly stretchable, so that the finished helical e-strip can stretch.
2. Recovery: it must have good recovery properties, returning to its initial length after stretching.
3. Compressibility: it must be compressible, to allow components facing the interior of the helical e-strip to compress into its surface, maintaining a smooth helical shape, and cushioning the components to provide support.

Rubber foam cord was identified as a good candidate, as it satisfies all of the above requirements. As there are several varieties of rubber foam cord, three of the most widely available options were selected for evaluation: neoprene, silicone, and ethylene propylene diene monomer (EPDM). These were subjected to mechanical tests to assess their suitability as helical e-strip core material.

3.1 Tensile test

A tensile test was performed, based on ISO 20932-1:2020+A1:2021[1]. A length of rubber foam cord was clamped in a Shimadzu AG-X Universal Testing Machine, such that the length between clamps was 10mm. The cord was then stretched to 50% extension 5 times, at a rate of 500 mm / minute. The resulting load was measured by the machine throughout the test. The average force at maximum extension was then calculated, and used to compare the cords. 3 mm and 5 mm diameter cords of each material were tested. After each test, the sample was removed from the testing machine, and its length was measured at 30 seconds, and again after 30 minutes, to assess recovery after stretching.

Tensile test results are shown in Supplementary Figure 2. Thicker cords of all materials required more force to stretch to 50%, as is expected, because thinner pieces of the same material will be inherently more stretchable. For 5 mm cords, EPDM was the most stretchable, though for 3 mm cords, EPDM and neoprene showed comparable stretch. However, recovery results shown in Supplementary Table 1 show that 3mm neoprene was significantly more deformed than the EPDM and silicone, when measured 30 seconds after the tensile test. All cords, of all diameters, did return to their original length by 30 minutes after the test. But as helical e-strips are designed to be embedded in stretch textiles, quick recovery is important. If a helical e-strip is embedded in a sports garment, the embedded electronics need to conform to the body, and not become temporarily deformed when the athlete kicks or throws a ball, or jumps, for example.

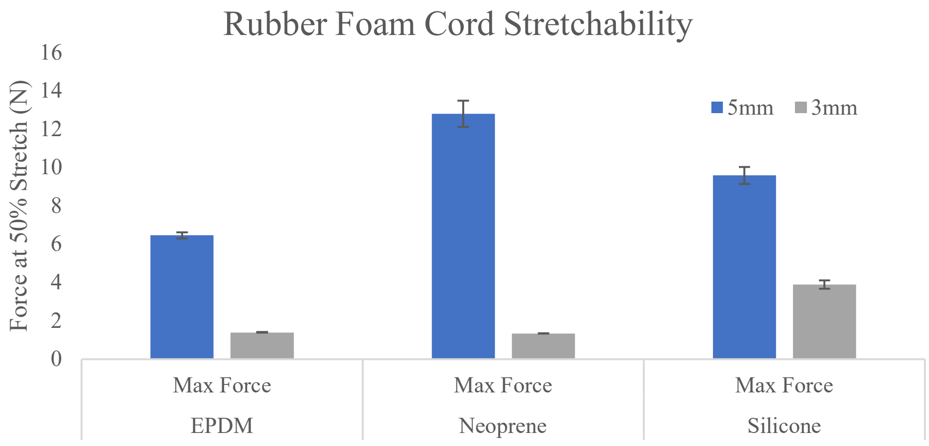

Supplementary Figure 3. tensile testing of rubber foam cords: EPDM, neoprene and silicone rubber foam cords, of both 3 mm and 5 mm diameter, were stretched to 50% elongation, 5 times. The average force at maximum elongation is shown here, averaged over 5 cycles.

Supplementary Table 1. Recovery properties of rubber foam cords: Cord length was measured 30 seconds after tensile testing, and % deformation calculated relative to cord's initial length. Cords were also measured 30 minutes after testing, to check whether they had returned to their original length.

Material	Diameter	% deformation 30 s after test	Recovery to initial length after 30 minutes?
EPDM	3 mm	6.49%	Yes
	5 mm	1.40%	Yes
Silicone	3 mm	2.07%	Yes
	5 mm	0.00%	Yes
Neoprene	3 mm	10.32%	Yes
	5 mm	0.00%	Yes

3.2 Compression test

A compression test based on ISO 7743:2011 [2] was carried out. Samples of each cord were placed between circular metal compression plates and compressed by 25% at a rate of 1 mm / minute. This was performed 4 times. Both 3 mm and 5 mm diameter samples were tested for each of the three rubber types. The results are shown in Supplementary Figure 3. Silicone cord of both diameters was the least compressible, and 3 mm EPDM cord was most compressible, with 5 mm EPDM and both diameters of neoprene in between.

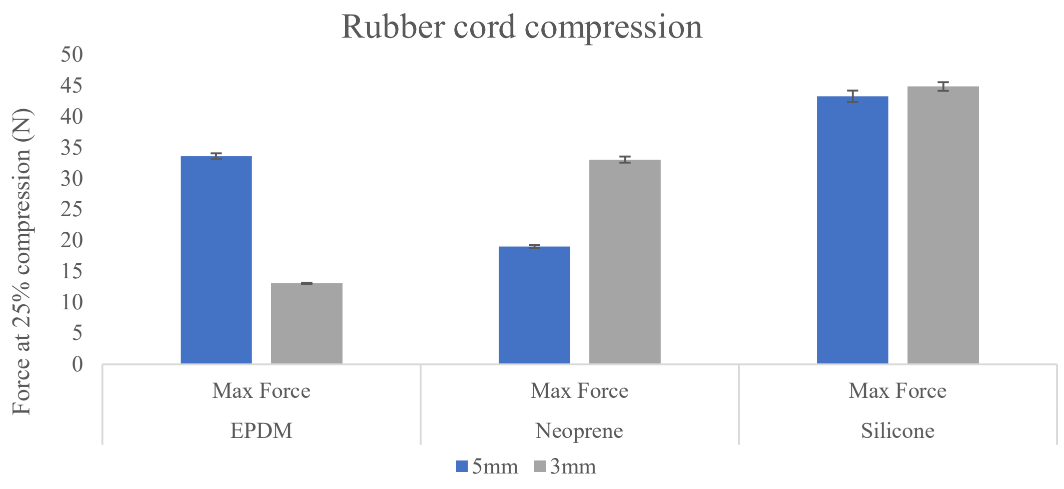

Supplementary Figure 4. Compression testing. 3 mm and 5 mm diameter EPDM, neoprene and silicone rubber cords were compressed up to 25% at a rate of 1 mm / minute.

3.3 Material choice

Other factors were also taken into consideration. The cost of cords was compared, with silicone foam cord being twice as expensive as EPDM and neoprene. All materials are available in medical grade, making them all suitable for medical e-textiles. EPDM and neoprene have high compatibility with

adhesives, whereas silicone is much more difficult to bond. Weighing these factors and the mechanical test results, EPDM was chosen as the core material.

The one exception to this is helical LED-strips, which needed to be transparent, and rubber foam cord was only available in opaque form. The selection of silicone tubing as a core material for LED-strips is covered in the main text in Sections 2.4 and 4.1.2.

4 Determining optimal geometry: width to diameter ratio

Supplementary Figure 5 illustrates the impact of component size on minimum *w* and *d*: if a component is too wide, or too tall, relative to the core diameter this creates irregularities in the helical geometry which is likely to reduce helical e-strip integrity and reliability. In this work, 0603 LEDs – with a footprint of 1.6 mm x 0.8 mm x 0.2 mm – were small enough that a planar e-strip containing them could be wrapped around a small diameter core (2-4 mm) without disrupting the helical geometry. But for larger components, such as temperature sensing or accelerometer integrated circuits, with footprints as in Supplementary Figure 5, it was clear that larger diameter cores would be required. This simplified description does not include solder and/or encapsulation materials. Therefore, the minimum diameter for a given component might be slightly larger than shown in the illustration.

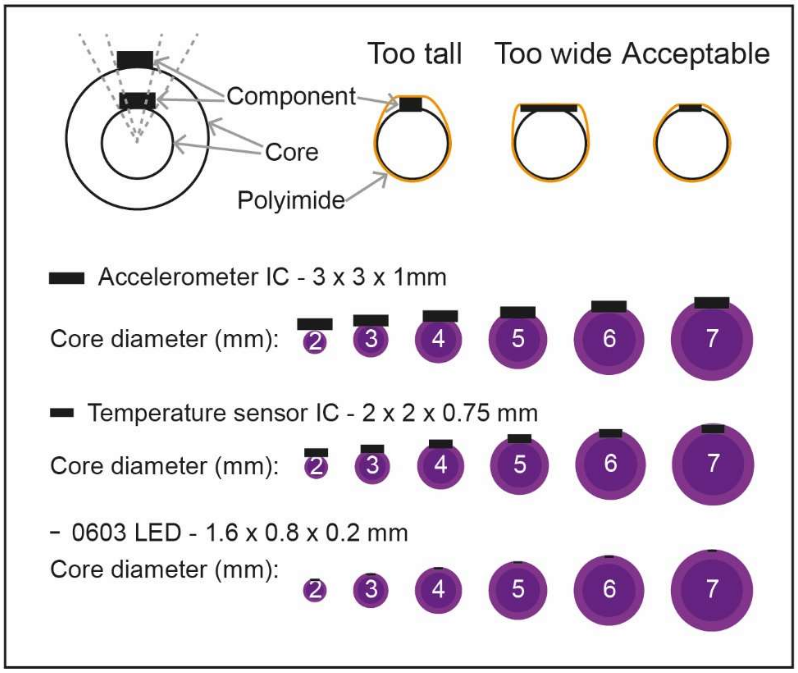

Supplementary Figure 5. Illustration of the constraints on w and d due to component size

Supplementary Figure 6 shows the results of a tensile test on blank helical e-strips with constant diameter and θ, and varying w, where three samples of each helical e-strip design were stretched until breaking point. The relationship between the width-to-diameter ratio and stretchability was not entirely linear, which may be because the helical geometry formed by manually winding the planar strip around the core. This led to some small irregularities in the helical geometry. Overall, reducing w, for fixed d and θ, resulted in increased stretchability. Reducing w increased s, the amount of exposed core not bonded under the planar e-strip.

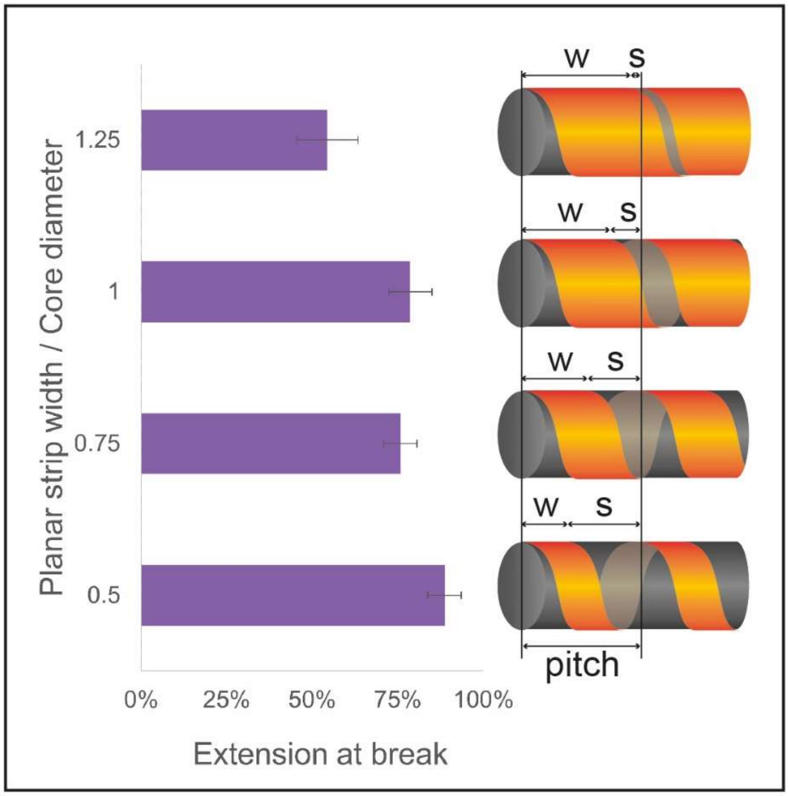

Supplementary Figure 6. Results of tensile tests on 4 mm diameter e-strips investigating relationship between stretchability and w:d ratio. Overall, a smaller w:d ratio results in higher stretch.

Based on this a lower $w{:}d$ ratio is desirable, but as already discussed, there are also constraints on w from the size of the components used. For a helical e-strip of 2 mm diameter, a $w{:}d$ ratio of 0.5 would require w =1 mm, which is difficult to achieve using SMD components. As mentioned in the main text, The smallest commercially available SMD package is 01005 (metric 0402), with dimensions of 0.4 mm x 0.2 mm, and due to its small size, this package is not widely used. And while the smallest components used in this work are less than 1 mm in size, a planar e-strip containing these components must be wider than the component itself, and may contain multiple conductive tracks which need to route around the component, which further increases the minimum width. For this work, a target ratio of $w = 0.7d$ was set, with an upper limit of $w \leq d$.

5 Wash testing data

Supplementary Figure 7 shows wash test data for each sample, after each wash cycle. An image of a full wash test sample is also shown.

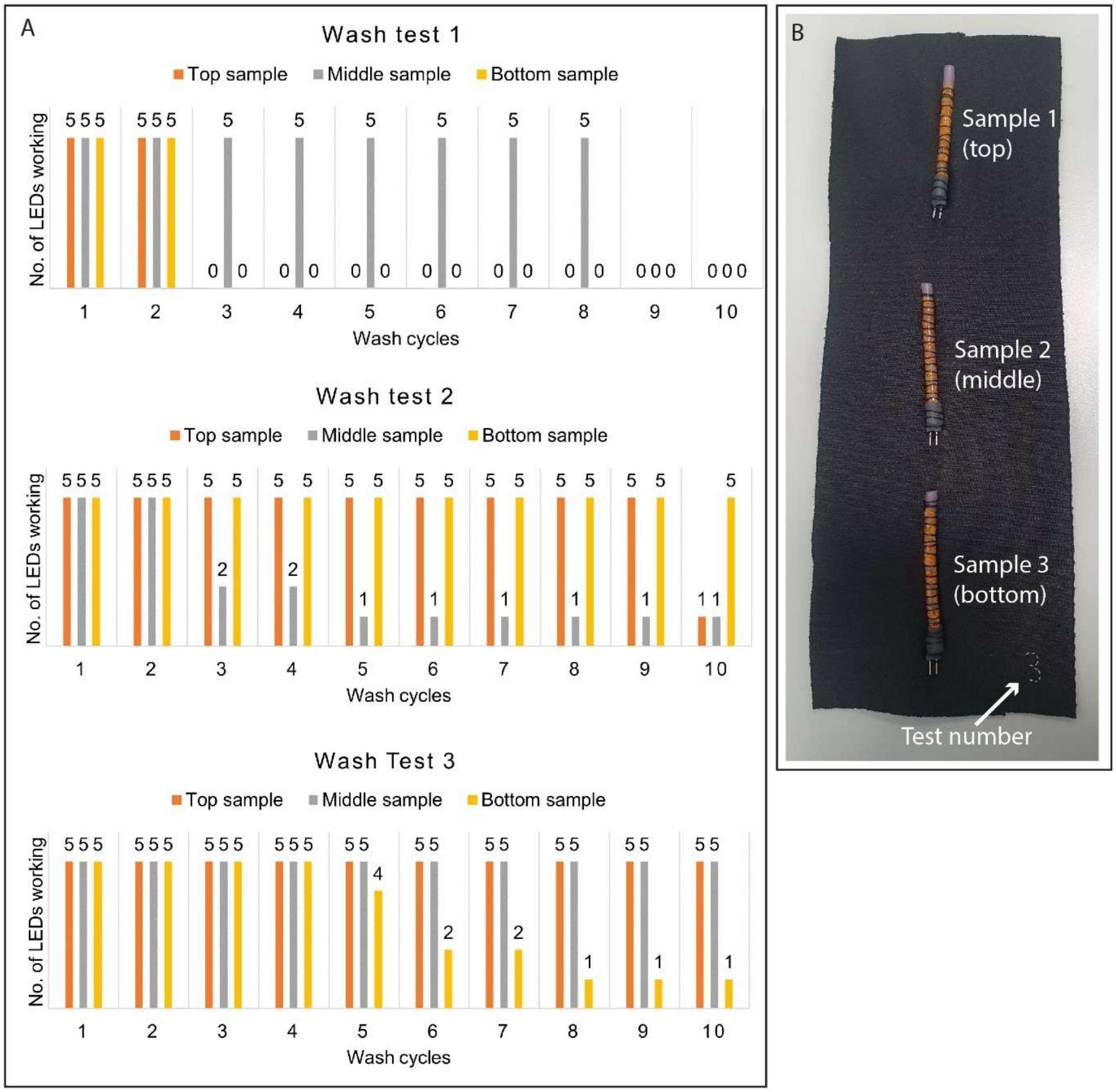

Supplementary Figure 7. Wash cycle testing: A) Results for each sample, after each wash cycle; B) Image of the wash testing sample used for Wash Test 3, with three helical LED-strip samples stitched onto a fabric piece.

6 Performance of the temperature sensing helical e-strip

Supplementary Figure 8 shows raw data from temperature sensing e-strips during each of the six test conditions. The planar e-strip data, in all conditions, shows small fluctuations in temperature. This is consistent with how the dry bath (hot plate) operates: it maintains a steady temperature, but within 0.3 °C, so there are small fluctuations. These fluctuations are most accurately recorded by the planar e-strip, but in the face down condition, the helical e-strips are also able to record the fluctuations (though to a lesser degree). In the side on condition, the helical e-strip data is more noisy, and there is no discernible effect of helical e-strip core diameter on the measurement accuracy. And in the face up condition, there is a further increase in noise and measurement error.

The test protocol involved maintaining the dry bath at a fixed temperature for 2 minutes, then increasing (or decreasing) the temperature by 2.5 °C, waiting for 2 minutes for the sensors to adjust to the new temperature, and then repeating. This can be seen clearly in Supplementary Figure 4. The response of the planar e-strip shows a faster response to the temperature changes, with the helical e-strips lagging behind, particularly in the side on and face up conditions. However, in the face down condition, this difference in response time is not considered an issue. 3 mm and 4 mm helical e-strips lag behind by less than 1 minute, and as the intended application is e-textiles, rapid fluctuations in temperature are not the main goal. A helical e-strip temperature sensing garment might, for example, monitor the temperature of an athlete during competition, with the aim of detecting early warning signs of heat stroke. Or monitor the temperature of a healing wound, to check for increases in temperature that might indicate the beginning of an infection. But these temperature changes occur gradually, rather than rapidly.

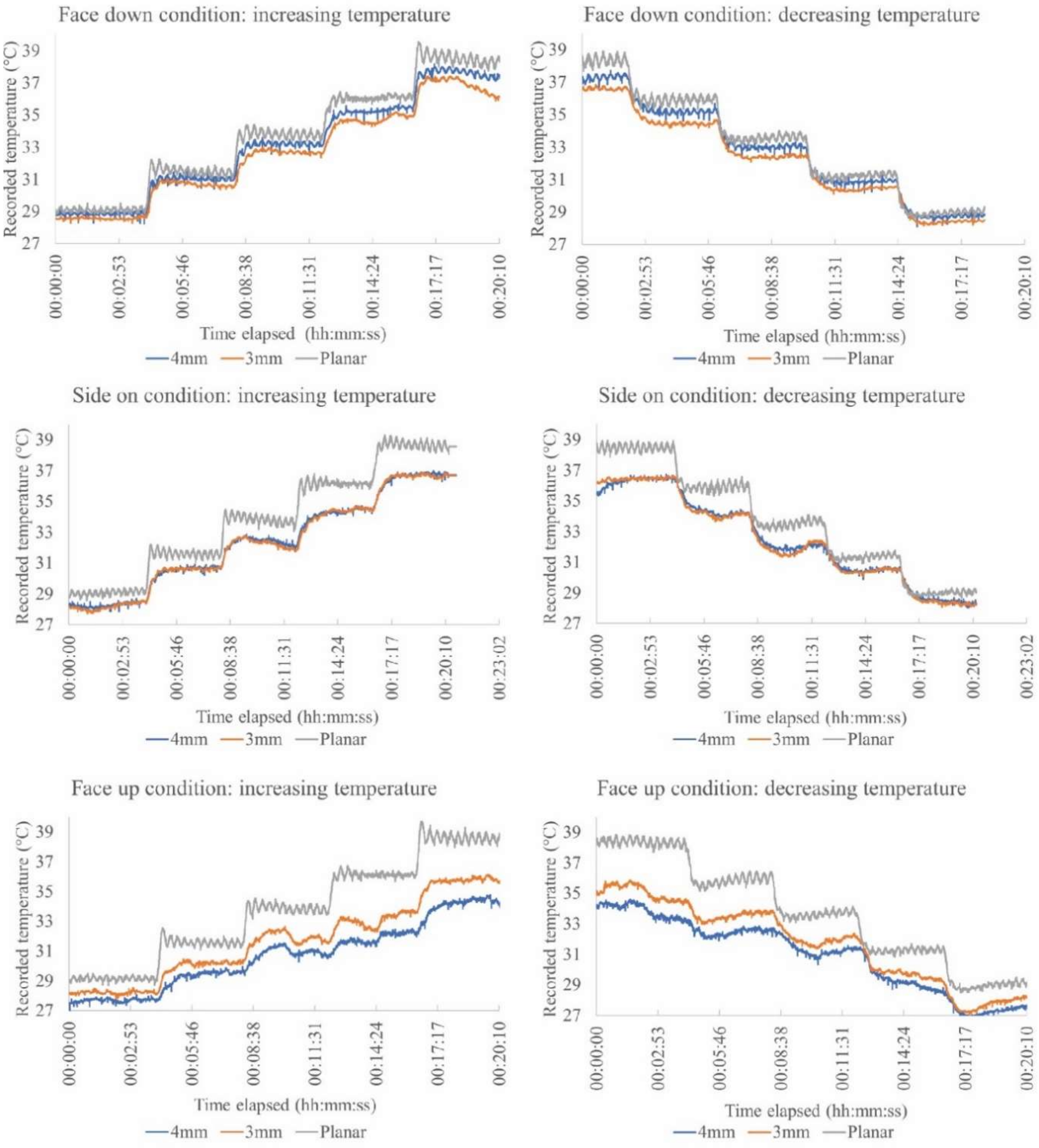

Supplementary Figure 8. Raw data from temperature sensing e-strips: Helical e-strips in the face down condition are most accurate, showing a slower response to temperature changes than the planar e-strip, but overall recording stable and relatively accurate values. In the side on condition, the diameter of the helical e-strip has no effect on the results, but the sensor readings are less accurate than in the face down condition. And in the face up condition, it is clear that the helical e-strip sensor measurements are noisy, and the least accurate of all three conditions.

7 References

1. International Organization for Standardization. ISO 20932-1:2018/Amd 1:2021 Textiles — Determination of the elasticity of fabrics — Part 1: Strip tests — Amendment 1. (2018).

2. International Organization for Standardization. ISO 7743:2011 - Rubber, vulcanized or thermoplastic - Determination of compression stress-strain properties. (2011).
